# A Raster-Based Methodology to Detect Cross-Scale Changes in Water Body Representations Caused by Map Generalization

**DOI:** 10.3390/s20143823

**Published:** 2020-07-09

**Authors:** Yilang Shen, Tinghua Ai

**Affiliations:** School of Resource and Environmental Sciences, Wuhan University, Wuhan 430072, China; yilangshen@whu.edu.cn

**Keywords:** land use change, water area, raster map, hierarchy, multi-scale

## Abstract

In traditional change detection methods, remote sensing images are the primary raster data for change detection, and the changes produced from cartography generalization in multi-scale maps are not considered. The aim of this research was to use a new kind of raster data, named map tile data, to detect the change information of a multi-scale water system. From the perspective of spatial cognition, a hierarchical system is proposed to detect water area changes in multi-scale tile maps. The detection level of multi-scale water changes is divided into three layers: the body layer, the piece layer, and the slice layer. We also classify the water area changes and establish a set of indicators and rules used for the change detection of water areas in multi-scale raster maps. In addition, we determine the key technology in the process of water extraction from tile maps. For evaluation purposes, the proposed method is applied in several test areas using a map of Tiandi. After evaluating the accuracy of change detection, our experimental results confirm the efficiency and high accuracy of the proposed methodology.

## 1. Introduction

The multi-scale representation of spatial data is a classic topic in cartography that requires many generalization operations, such as simplification, aggregation, typification, and displacement [1,2,3,4,5,6], to be realized. Water is necessary for economic, social, and environmentally sustainable development. For the multi-scale representation of water areas, generalization operations such as the selection of objects, the simplification of boundaries, and the aggregation of multiple objects are usually applied, which leads to changes in water areas in terms of the quantity, shape, and distribution. To solve the quality problem of multi-scale maps, a change detection method can usually be applied.

Change detection refers to the process of identifying differences through a comparative analysis of the state of the same geographic entity acquired from images or databases in different periods [7], and it is an important method to maintain spatial data and update spatial databases [8,9]. Consistent multiresolution spatial data are required in many applications, such as rapidly transmitting spatial data over the internet [10], querying multi-resolution spatial data [11], and extracting and integrating spatial data information with different levels of detail [12].

On one hand, the traditional spatial data change detection methods ignore the influence of cartographic generalization, which cannot be applied for the change detection of cross-scale water body representations. For example, many scholars have detected changes in water systems at a single or adjacent scale, such as lake change detection [13,14,15], river change detection [16,17], coastline or coastal zone change detection [18,19,20], and water system [21] change detection. In addition, some mature software programs such as Map Comparison Kit [22] were developed to compare the differences of different raster maps. However, different generalization operators, such as simplification, smoothing, aggregation, omission, and displacement, are used to generate spatial data at a lower resolution from data at a higher resolution [23]. Therefore, multi-resolution spatial data always contain inconsistencies in topological, directional, and metric relations due to the measurement methods, data acquisition approaches, and map generalization algorithms. The methods of water change detection at a single scale are not appropriate at multiple scales. As a consequence, a mechanism is needed to check these changes.

On the other hand, most of the literature on water system change detection uses time series of remote sensing image data. The technology of water change detection based on remote sensing images is relatively mature, and the process is relatively fixed. Relative to remote sensing images, the technology for change detection based on vector data is much scarcer. In addition, some scholars have tried to use both remote sensing images and vector data to detect changes in spatial data [24]. However, under the background of big data, the data sources and types of GIS vary. The problematic issue is how to extract meaningful information from a growing number of unstructured data types (such as pictures, street views, 3D models, and videos) [25]. For example, the source tile map from OpenStreetMap data usually contains data quality problems [26,27], and the data inconsistency generated from map generalizations at different scales often exists due to the contribution of a large number of volunteers [28,29]. Checking the inconsistency problem is meaningful to improve the quality of the map data. Current studies of water change detection cannot deal with multi-scale changes involving the shape, location, number, orientation, and size of objects from different data sources, such as tile maps.

Therefore, in this paper, a new method for multi-scale water area change detection based on raster maps is developed. From the perspective of spatial cognition, we propose a theory of the hierarchical detection of water area changes in multi-scale raster maps. The water area change situations are classified into several new categories. According to the different types of water area changes, we establish a set of detection indicators and water area rules for change detection, and finally, the change detection accuracy is evaluated.

This paper is organized into five sections. Section 2 presents the hierarchical principles of water change detection at multiple scales, including three hierarchies: the body level, the piece level, and the slice level. Section 3 presents the types of multi-scale water area changes. We propose the rules of change identification and the hierarchical methods of expressing change information. Section 4 presents the data set and shows the experimental process and results. The experimental results indicate that our method is feasible and effective.

## 2. Hierarchical Change Detection

The hierarchy of multi-scale spatial data change is divided into three layers: the body layer, the piece layer, and the slice layer. The body layer characterizes the global changes in multi-scale data, the piece layer characterizes the mutations of multi-scale data, and the slice layer focuses on the detailed changes between two adjacent scales.

The structure of the hierarchy is similar to that of a tree and is used to organize and structure complex systems. A system can be divided into smaller subsystems, and the subsystem can be further divided into smaller subsystems. A hierarchy can be formed in this circulatory way [30]. In geographic information science, some scholars have applied hierarchy to geographic space division, geospatial data storage and display, and many other aspects [31,32,33].

The notable cognitive linguist Langacker once said, “Hierarchy plays a very important role in human being’s spatial cognition [34].” A human being’s conceptual model of reality demonstrates significant hierarchical spatial characteristics. During mental processes, humans usually do not consider the whole problem with all of its details; rather, they tend to divide the problem into several parts or simplify it while neglecting its details and then investigate these details in depth and hierarchically. This kind of technique for solving the problem by starting with the general scope and then focusing on the details, or by starting from the whole and moving to the partial, is the so-called hierarchical method. Change is a kind of difference information in spatial data. This paper argues that people should consider hierarchical features in the process of acquiring regularity; then, they can be aware of the essence of change information from simple to complex scales and better master the change rules of the spatial data from the macro to micro scales. Using hierarchical change detection, we can obtain the right amount of change information at different scales and avoid cognitive difficulties.

### 2.1. Division of Detection Hierarchy

To see details and focus their attention on the main problem without interfering with minor issues, people always hierarchically abstract subjects in an orderly way when thinking. As the level of detail exceeds a certain degree, the more things a person can see, the less things they can express [35].

The detection of detailed change information shows that we detect spatial change information between two uniform or adjacent scales. However, to better grasp the global change situation of spatial data, we need to observe and analyze changes in multi-scale spatial data from the macro view. Hence, this article attempts to study multi-scale spatial data changes at different spatial scales.

### 2.2. Body Layer

Spatial entities within the same scope have different detailed expressions at different map scales. Multi-scale spatial data can be seen as a collection of data composed of a group of spatial objects on the order of different map scales. This collection of data can be called a spatial series. Each object of the spatial series corresponds to an expression of the spatial entity at a certain map scale. A spatial series of the same geographical object can be expressed mathematically:

(1) $$X_{1},X_{2},\cdots ,X_{n}$$

This expression can be abbreviated as $\left\{X_{n},n\in N\right\}$.

Similar to the relationship between the samples and the sample observations, we can use Formula (2) to express *n* ordered sets of observations of the spatial series in Formula (1). Formula (2) can be called the observation series of length *n*:

(2) $$x_{1},x_{2},\cdots ,x_{n}$$

In the body layer, we analyze and research the change information of the spatial data from macroscopic and global perspectives, and the rules or information included in the orderly observations $x_{1},x_{2},\cdots ,x_{n}$ of the geographical objects are discovered.

For example, when the observed value x represents the area of a lake, namely, $x_{n}=Area_{n}$, through the variance calculation of this set of observations {$Area_{1},Area_{2},\cdots ,Area_{n}$}, we can obtain the dispersion degree of area change in a lake in a multi-scale map. If the variance D(Area) is relatively large, the area of the lake would have a high dispersion degree and noted fluctuations in *n* scales. If the variance D(Area) is relatively small, the area of the lake would have a low dispersion degree and small fluctuations in *n* scales.

### 2.3. Piece Layer

In the map expression space, the scale range of every spatial entity is limited [36]. Taking a building as an example, buildings are usually expressed in the form of a polygon in large-scale maps, a simplified rectangle in medium-scale maps, and a point on small-scale maps. On maps with a scale of 1:250,000 or smaller, buildings can no longer be expressed unless they are special. This kind of change, with a substantial difference from a polygon to a rectangle or from a rectangle to a point, is called a mutation in this paper. In the piece layer, the mutation information is detected and expressed.

For a set of spatial series $X_{1},X_{2},\cdots ,X_{n}$, the purpose of detection in the piece layer is to group this set of series, which makes the differences obvious among different groups, while the differences are less obvious among objects in one group. For example, for a set of spatial series {$X_{1},X_{2},X_{3},X_{4}$}, if the difference $\Delta_{12}$ between object $X_{1}$ and object $X_{2}$ is relatively small, and the difference $\Delta_{34}$ between object $X_{3}$ and object $X_{4}$ is also relatively small, while the difference $\Delta_{23}$ between object $X_{2}$ and object $X_{3}$ is relatively large, then $X_{1}$ and $X_{2}$ can be classified as one group, and $X_{3}$ and $X_{4}$ can be classified as one group. Therefore, in the process of piece layer detection, we should focus on the larger difference $\Delta_{23}$ between the groups instead of the smaller difference $\Delta_{12}$ or $\Delta_{34}$ in one group.

**Figure 1:**
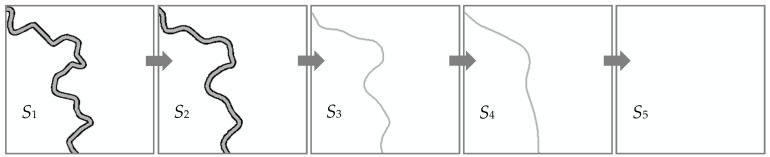
Mutation change in the piece layer.

As shown in Figure 1, the change between $S_{1}$ and $S_{2}$ is due to the process of boundary simplification of the double river. This kind of change is a slow change. The change after $S_{2}$ is a process of transforming the geometrical dimension, which can be seen as a mutation. The change between $S_{3}$ and $S_{4}$ is due to the process of shape simplification of a single river. This kind of change is a slow change. After $S_{4}$, the river disappears, which can be seen as a mutation again. In the piece layer, we focus on how to detect the mutation change between $S_{2}$ and $S_{3}$ instead of the slow change between $S_{1}$ and $S_{2}$. The reason is that the mutation change produces more differences than slow changes.

### 2.4. Slice Layer

After grouping a set of spatial series $X_{1},X_{2},\cdots ,X_{n}$ in the piece layer, the difference between adjacent scales in each group is smaller. Relative to the piece layer, in the slice layer, this kind of change information is detected in every “piece” with a smaller difference. For example, in the spatial series {$X_{1},X_{2},X_{3},X_{4}$} in Section 2.3, $X_{1}$ and $X_{2}$ are in one group, and the difference $\Delta_{12}$ between $X_{1}$ and $X_{2}$ is smaller. In the slice layer, this kind of change with a smaller difference, such as $\Delta_{12}$, is handled.

**Figure 2:**
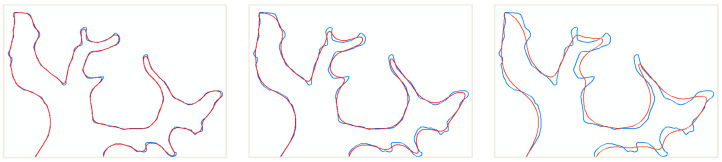
Detailed change in the slice layer.

In the process of map generalization [37,38,39,40,41,42], boundary simplification [43,44,45] usually produces changes in the slice layer. As shown in Figure 2, a famous simplification algorithm [46] is used for the smoothing curve. Figure 2 shows the smoothing results (red lines) of originalboundaries (blue lines) with different tolerances. This kind of detailed change with a smaller difference in Figure 2 is a slice layer change.

## 3. Change Classification and Detection of Water Area

It is necessary to consider the change type in change detection time. In the past few years, scholars have classified the change type from different perspectives depending on the type of object. Claramunt subdivided the evolution of a single entity into eight types: appearance and disappearance, movement, rotation, expansion, shrinkage, and deformation [47]. Raza and Kainz treated land blocks as the analysis object and subdivided the change types of areal objects into eight types: appearance, disappearance, size change, shape change, movement, transformation, merging, and splitting [48]. The simplest outcome for change detection is straightforward; that is, the target data are either changed or not [49]. However, it is necessary to take the influence of map generalization into account when we detect changes in water objects at multiple scales. The existing classification methods do not consider the problem of scales, and they are not appropriate for the change detection of water objects at multiple scales.

There are two kinds of reasons for water changes at multiple scales. First, map generalization can cause differences between different scales. Water systems can be divided into two types without considering the type of water: linear water and areal water. Linear water includes a single river system and a double river system. The generalized operation of the former includes the river selection number and chooses what and how to simplify the system. The generalized operation of the latter includes the extraction of the double river central axis and the simplification of a single river after extraction. Areal water includes lakes and reservoirs. The generalized operation of areal water includes selection and simplification. Second, the differences caused by the actual measurements may lead to water changes at different scales.

By analyzing the causes of changes, the change types of water areas at multiple scales are divided into 9 categories in this paper, for a total of 14 subtypes, on the basis of existing change types. For all of the change types, we establish a set of indicators and rules to detect them and use hierarchical thought to classify and express the change information in three layers: the body, piece, and slice layers.

### 3.1. Definition of Detection Indicators

The types of water area change are diverse. It is difficult to identify and differentiate the various change types when we only use some basic indicators of geometric objects, such as length, area, shape, and center. For the purpose of detecting and inferring the change types of water areas, we define a series of new indicators for identifying them, and those different indicators are combined for use in this paper. These inference indicators include the following nine types: matching type, length, area, shape index, geometric center, overlap degree, buffer overlap degree, geometric type, and hole state. The calculation methods of these indicators are as follows:

Matching type *MT*: The matching types of an object area mainly include five kinds: MT=1:0, MT=0:1, MT=1:1, MT=1:m, and MT=n:1, in which *m*, *n*, 0, and 1 represent the number of objects.

Length *L*: The length is used to describe the boundary of the water area. Before the length of the object is calculated, it is necessary to extract the edge pixels of the object area. Then, the length of the water object is calculated by counting the number of pixels *Num*, namely, L=Num. The length difference $\Delta_{L}$ before and after the change can be represented as $\Delta_{L}=\left|L_{after}-L_{before}\right|$.

Area *A*: The precise area of the region is calculated by counting the number of pixels *Num*, namely, A=Num. The area difference $\Delta_{L}$ before and after the change can be represented as: $\Delta_{A}=\left|A_{after}-A_{before}\right|$.

**Figure 3:**
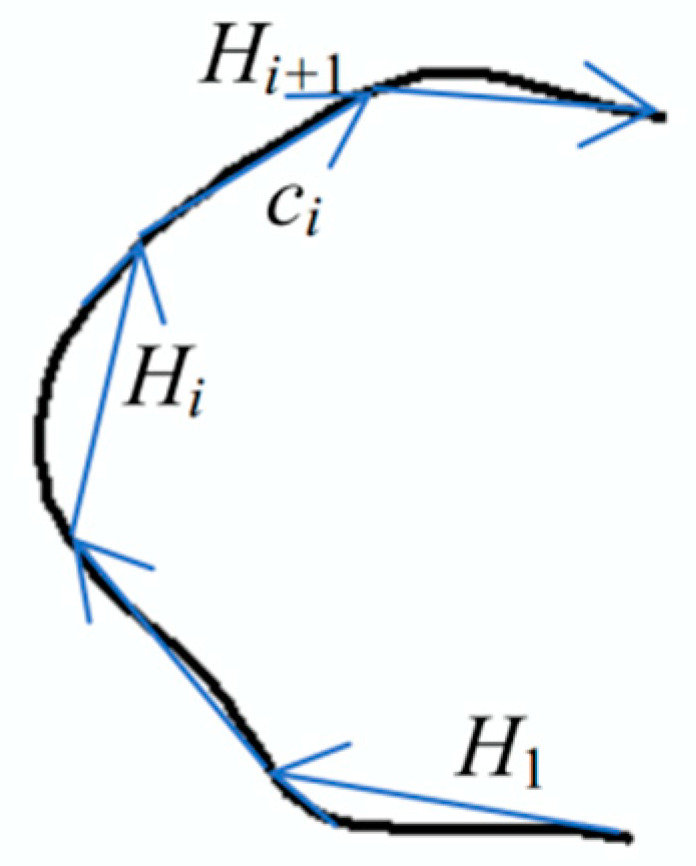
Included angle chain method.

Shape index *SI*: The shape index is used to describe the shape of a linear object or the boundary of an areal object. The common methods to describe shape and calculate similarity include Fourier descriptors [50,51] and the angle difference integration method [52]. In this paper, the included angle chain method [53] is used to describe the shape and calculate the similarity of the linear object or the boundary of the areal object. In this method, as shown in Figure 3, a curve is modelled by a number of linked equi-length line segments, for example, a length of 50 pixels at levels 11 and 12 in the experimental section and a sequence of codes using the included angles between a pair of neighboring line segments are used to represent the curve. The representation is invariant to rotation, scaling, and translation [53]. The Euclidean distance of the included angle chain code in *n*-dimensional space can be used to measure the similarity between matching objects.

Suppose that the set of equi-length line segments of curve *Q* is $\left\{c_{1},c_{2},\cdots ,c_{n}\right\}$; then, the included angle chain code of curve *Q* can be denoted by *A*, and *A* is defined as shown below:

(3) $$A=\left\{\alpha_{1},\alpha_{2},\cdots ,\alpha_{n-1}\right\}$$

in this equation, $\alpha_{i}$ denotes the angle from line segment $c_{i}$ to line segment $c_{i+1}$ in the counter-clockwise direction. The shape difference $\Delta_{SI}$ before and after the change can be represented as

(4) $$\Delta_{SI}=\frac{\sum_{i=1}^{n}\mathrm{dif}\left(\alpha_{i}^{after},\alpha_{i}^{before}\right)}{n}$$

in this equation $,dif\left(\varphi_{1},\varphi_{2}\right)=\{\begin{array}{c} \left|\varphi_{1}-\varphi_{2}\right|,\;if\left|\varphi_{1}-\varphi_{2}\right|\leq \partial \\ 2\partial -\left|\varphi_{1}-\varphi_{2}\right|,\;if\left|\varphi_{1}-\varphi_{2}\right|>\partial \end{array}$, *∂* is a set threshold.

Because the angle number of two linear objects may be different, for example, linear object *A* with *n* angles and object *B* with n+m angles, in the actual calculation, by combining other detection indicators, we only need to calculate $\Delta_{SI}$ using the first *n* angles with the same starting point and counter-clockwise direction to meet the identification requirement.

Geometric center *GC*: The geometric center of the irregular polygon is usually equal to its center of gravity when its density is uniform, and the location of the center of gravity is only related to the shape of the object. Assuming that *f*(*i*, *j*) is a matrix that represents an image, in which *i* and *j* are the row and column values of this matrix, respectively, the coordinates of geometric center $GC\left(i_{c},j_{c}\right)$ can be calculated as follows:

(5) $$\begin{array}{cc} i_{c}= & m_{10}/m_{00},\;j_{c}=m_{01}/m_{00},\\ m_{pq}= & \sum_{j=1}^{N}\sum_{i=1}^{N}i^{p}j^{q}f\left(i,j\right) \end{array}$$

The difference in geometric center $\Delta_{GC}$ before and after the change can be represented as $\Delta_{GC}=dis\left(GC_{after},GC_{before}\right)$, where *dis* is the Euclidean distance between two points.

**Figure 4:**
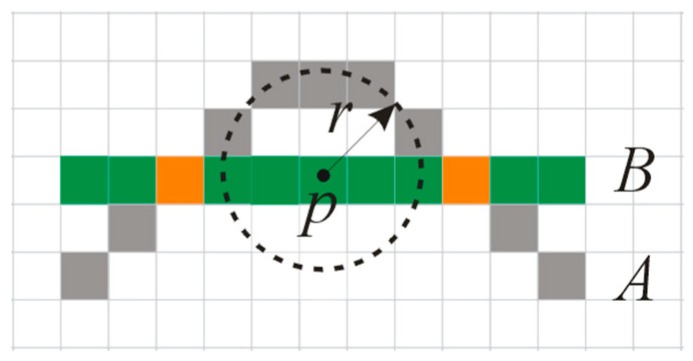
Overlap degree.

Overlap degree *OD*: For each group of matching water area objects, the overlap degree *OD* is the ratio between the overlapping pixel number $Num_{lap}$ and the total pixel number $Num_{all}$ after overlaying, namely, $OD=Num_{lap}/Num_{all}$. As shown in Figure 4, curve *A* is composed of 11 pixels, curve *B* is composed of 11 pixels, and the overlapping portions between curves *A* and *B* appear red. It is easy to see that the overlap degree of *A* is $OD_{A}=Num_{lap}/Num_{all}=2/11=18.2$%, and the overlap degree of B is $OD_{B}=Num_{lap}/Num_{all}=2/11=18.2\%$.

As shown in Figure 4, after overlapping, when the pixels of one object *A* are in the buffer of pixel *p*, *p* is one pixel of object *B*, and then pixel *p* is also seen as an overlapping pixel, even if there is no pixel overlapping with *p* in object *A*. Suppose that the total overlapping pixel number of matching objects is $Num_{lap}$; the total pixel number of matching objects is $Num_{all}$, and then the buffer overlap degree of the matching objects can be expressed as $BOD=Num_{lap}/Num_{all}$. In Figure 4, when the buffer radius *r* is equal to 3 pixels, the buffer overlap degree of curve *A* and curve *B* is $BOD=Num_{lap}/Num_{all}=22/22=100\%$. For example, at levels 11 and 12 in the experimental section, the value of *r* is equal to 40 pixels. 

Geometric Type GT: The geometric type of a water object is classified into linear and area objects. When the object l is linear water, $GT_{l}=0$, and when the object l is a water area, $GT_{l}=1$.

Hole State HS: When there are no holes in the water area, HS=0; otherwise, HS=1.

Before the identification of change types, some simple operations, such as moving, scaling, and overlaying, may be applied to the water objects to distinguish all of the change types. In addition, the parameter values used in the actual experiment are not fixed. The selection of these parameter values depends on the image resolution and different application scenarios.

### 3.2. Change Types and Detection Rules

#### 3.2.1. Change Types

In this paper, the change types of water areas are divided into 10 categories, for a total of 15 subtypes, as shown in Table 1. The 10 categories are no change, appearance, disappearance, movement, rotation, scaling, boundary generalization, morphology change, derivative change, and hole change. Among them, the morphology change includes appending, distribution, and shape changes; the derivative change includes merging and splitting; and the hole change includes appearance, disappearance, and structure change of the hole.

#### 3.2.2. Detection Rules

We define a series of detection rules to detect the change types of water areas at multiple scales. The detection rules are made up of operations and indicators. For each type of change, some operations, such as moving, scaling, or overlaying, may be used to detect it. Then, several of the nine indicators are selected and combined. Each change type corresponds to an indicator combination. Table 2 shows the detection rules of all 15 water area change types presented in this paper.

When identifying a certain change type, not all of the indicators need to be used. Therefore, the indicators are divided into relevant indicators and irrelevant indicators. The relevant indicators are used to identify the change types, and the irrelevant indicators are ignored. The relevant indicators are divided into threshold values and fixed values. Suppose that the matching objects are *x* and *y*; then, the detection rules of all change types are detailed in Table 2.

#### 3.2.3. Hierarchical Expression of Water Area Changes

**Figure 5:**
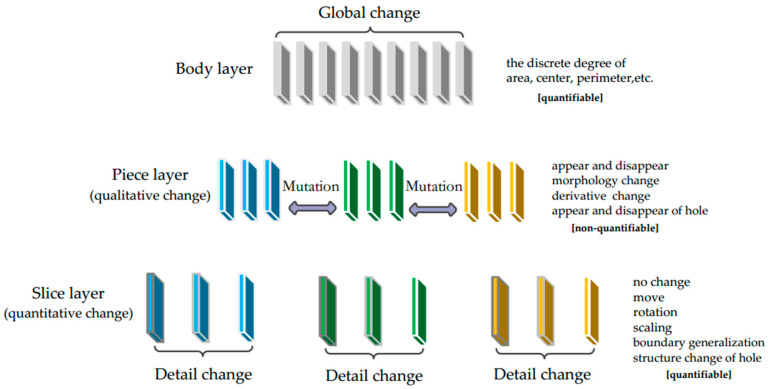
Hierarchical expression of water area changes.

For the changes in every water object area at multiple scales, we establish a form of hierarchical expression for displaying the detection results. As shown in Figure 5, in the body layer, by analyzing the discrete degree of the area, the perimeter, or the center of the water area, the global fluctuations in the changes in the water area at multiple scales can be expressed. The metrics of global fluctuation can be the maximum deviation, the mean deviation, the standard deviation, and so on. In the piece layer, the mutation information of the water area at multiple scales is expressed. The corresponding mutation types include appearance, disappearance, morphological change, derivative change, and the appearance or disappearance of holes. It is difficult to use quantitative indicators to measure the differences in the mutations. Therefore, in this layer, the mutations can be seen as qualitative changes.

In the slice layer, the detailed change information of the water area at multiple scales is expressed. The corresponding change types include no change, movement, rotation, scaling, boundary generalization, and structural change of the hole. It is easy to use quantitative indicators to measure the differences in the detailed changes. Therefore, in this layer, the changes can be seen as quantitative changes. Among them, the difference in the movement change can be measured by distance, the difference in the rotation change can be measured by the angle, the difference in the scaling change can be measured by the ratio, the difference in boundary generalization can be measured by shape similarity, and the difference in the structure change of the hole can be measured by length or area.

In other words, from a cognitive rule perspective, the hierarchical methods proposed in this paper are used to detect and express the changes in water area at multiple scales (from global to local, from macro to micro, and from qualitative to quantitative), which enables us to grasp the essence of change information from rudimentary to profound and better master the laws of changes at multiple scales.

## 4. Experiments and Analysis

### 4.1. Water Area Extraction and Reconstruction

How to extract water from tile elements is a problem that cannot be ignored. Therefore, before change detection, we focus on how to extract independent water areas from the tile map. Compared with the colors of the other elements in a map, water areas are usually expressed with a uniform blue color. Therefore, it is effective to separate water areas from other geographical elements on one map when using a given color threshold. However, due to the overlay of other elements on the map, such as roads and annotations, the extracted water areas contain obvious fractures or hollow phenomena. In addition, the boundary colors of the water areas are usually not the same color but have gradual change effects. As a result, the boundary of the water areas needs to be processed specifically. Considering these two factors, this paper uses the following steps to extract water areas from tile maps [9].

(1) Extract water areas with color segmentation

**Figure 6:**
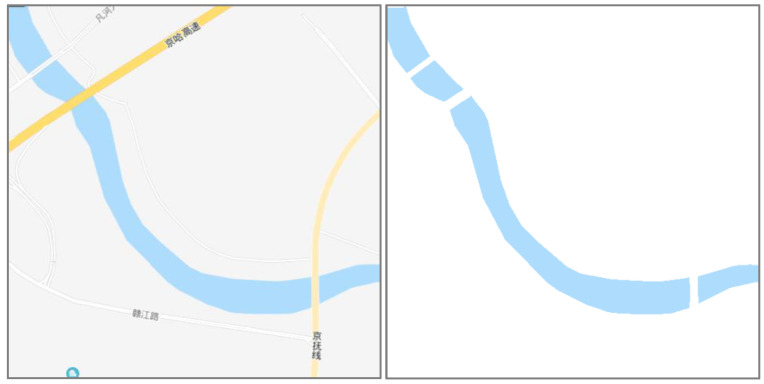
Extracted water areas with color segmentation.

The water colors of different tile maps are different. However, the colors of water in one map are uniform. Therefore, it is effective to separate water from other geographical elements in one map when using a given color threshold. As shown in Figure 6, the left figure is a tile map of Tiandi, and the right figure is the result after extraction by using the color threshold. However, this extraction method is not suitable for scanned paper maps with various color values. Due to the overlay of the roads, the extraction area is not continuous, but there is a certain fracture.

(2) Fracture connection of water areas

**Figure 7:**
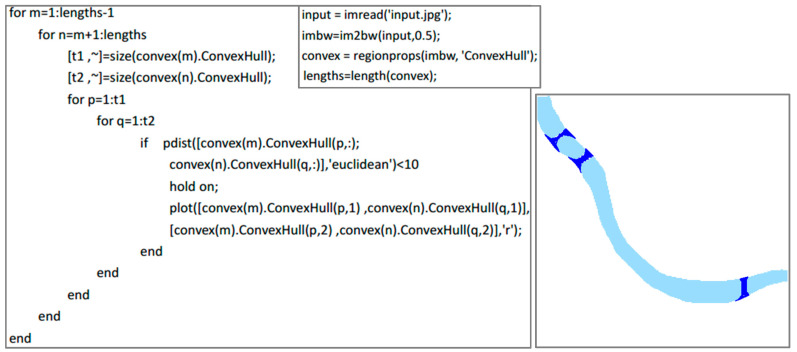
Fracture connection of the water areas.

Due to the overlay between water and other geographical elements on the map, such as roads, water areas may contain fracture phenomena. As shown on the left side of Figure 6, the roads overlay the river, which leads to the fracture phenomenon in the extracted river. To connect the fracture, this paper uses an algorithm based on a convex hull to connect the fracture. The basic principle of this algorithm is shown below. First, the convex points of each connected domain should be calculated. Then, when the distances between convex points from two connected domains are less than a given threshold, these convex points should be connected. Finally, the connection lines should be converted into pixels to generate new rivers. The code is shown on the left side of Figure 7. The algorithm is based on the MATLAB environment, and the meanings of the corresponding functions and variables can be found in the MATLAB official help documentation. The right side of Figure 7 is the result after using the algorithm to connect the fracture. It was found that the algorithm maintains the original width and tendency information of the river well.

(3) Hole filling of the water areas

**Figure 8:**
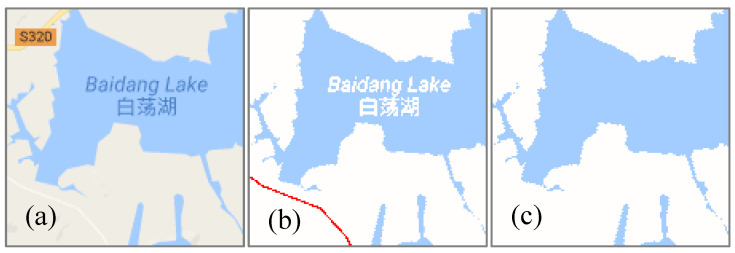
Hole filling of the water areas. (**a**) the original water areas, (**b**) non-feature pixels, (**c**) the results of filling holes.

For some larger planar water areas, because of the overlay of features, the extraction areas may contain holes, as shown in the middle of Figure 8. To solve this problem, it is necessary to determine if the neighborhoods of the feature pixels, such as the dark blue annotation pixels in Figure 8a, contain water pixels. Pixels with other inconsistent colors will be considered as non-feature pixels and finally removed, such as the red road pixels in Figure 8b. Assuming that the width of a neighborhood is 2 pixels, and it belongs to eight connected types, if the neighborhoods of the feature pixels contain water pixels, then the feature pixels are marked as water pixels. Otherwise, the feature pixels are not marked. In an actual experiment, a relatively high accuracy can be realized when the width is set to 2 or 3 pixels. The Figure 8c is the result of using this method to fill holes. Although this method of hole filling of the water areas is simple and limited, it can satisfy the basic experimental requirements because the size and color of features on the same map, even in different areas, are usually consistent.

In addition, for a general raster map, the matching of water areas can be realized according to the pixel coordinates of the same features. For a tile map in this experiment, the water areas at different levels of detail are matched by overlaying operations. Because a tile from a map at a high level is always divided into four tiles with the same size at an adjacent low level, overlapping water areas can be treated as matched objects by twofold magnification.

### 4.2. Study Area

In this paper, the experimental data used to detect changes in water areas at multiple scales come from the map of Tiandi [54], China. Because the lakes basically have no changes before the 7th level and after the 13th level in the Tiandi map, we chose the data with the same map extent from the 7th (1:5,000,000) to the 13th (1:100,000) level of the Tiandi map; the detailed scales of the levels between the 7th and 13th levels can be found on the official website [54]. The area is located in western Qinghai Province, China. There are many lakes and few rivers in this area. The range of the area is between 34.34° and 36.62° N latitude and between 89.97° and 92.87° E longitude. The data at the 7th level consists of a piece of tile, and the size and coordinates of this tile are 256 × 256 pixels and x=96,y=50, respectively. With the increase in level, the number of tiles also increases in accordance with pyramid rules. The data in the highest level, the 13th level, consist of 4096 tiles. The coordinate range of those tiles is 6144≤x≤6207 and 3200≤y≤3263. The size of the image consisting of those tiles is 16,384 × 16,384 pixels.

**Figure 9:**
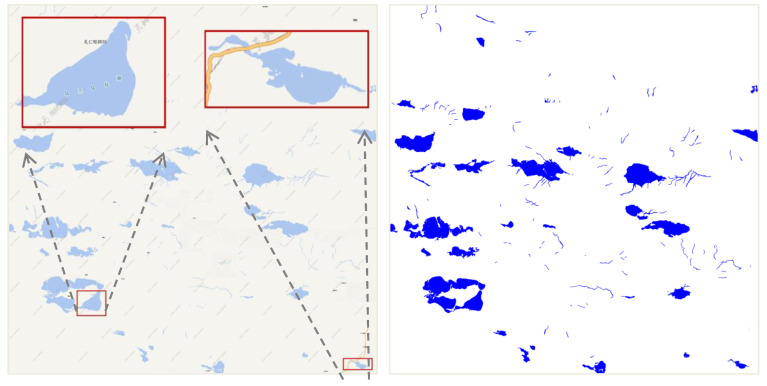
The experimental data.

Figure 9 shows the tiles in the 11th level. The left panel shows the original tile data, from which we can see the overlay phenomenon between the lake and the roads or features. The image on the right shows the situation after extraction. We find that the results after fracture connection and hole filling are good.

### 4.3. Result and Analysis

(1) Detection of change types

According to the proposed hierarchical detection method, after multi-scale target matching, we detect the changes in the study area by calculating the corresponding indicators and using certain operations. The calculation method of each indicator is shown in Section 3.1, and the detection rule of each change type is shown in Section 3.2. Table 3 shows the results of detection.

Table 3 shows that from level 7 to level 10, the change types and number of changes are lower. The main change types are boundary generalization, morphology change, and hole change. There are no other change types. From level 10 to level 12, the change types and number of changes increase significantly. The main change types are appearance, disappearance, morphological change, derivative change, and hole change. Among them, the proportion of appearance changes is the highest, accounting for 56.84% of all changes. The proportions of derivative change and hole change are second, accounting for just 5.6% and 3.1%, respectively. The proportions of disappearance and morphological changes are the lowest, accounting for only 1.1% of all changes. However, the proportion of disappearance depends on the direction of scale change. From level 12 to level 13, the change types and number of changes decrease significantly. Among them, the proportion of appearance change is still the highest. The proportion of hole change is the second highest. In general, the number of changes is smaller from level 7 to level 10. The number of changes increases significantly from level 10 to level 12 and decreases significantly from level 12 to level 13. Of all the change types, the appearance change is the greatest, accounting for 84.8% of all changes. The proportion of hole changes is the second highest, accounting for 8.1% of all changes. The numbers of morphology change and derivative change are lower; together, they only account for 5.8%. The remaining detected change types disappear and are identified as boundary generalization changes. In addition, some other change types, such as moving, rotation, scaling, derivative change 1, and hole change 2, are not detected. To verify the validity and reliability of our method, we checked all 15 change types by overlaying and visual judgement methods and found that the accuracy of change type detection is 91.1%. Mainly, the wrong types come from the morphology and hole changes. In addition, as the extraction of water areas is simple and limited, many detection errors occur to a certain degree.

**Figure 10:**
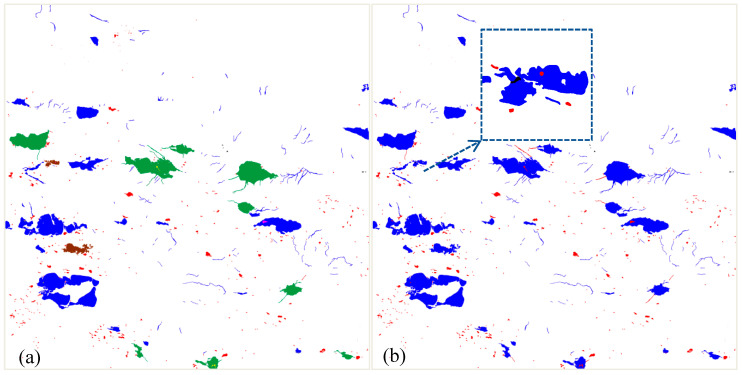
Results of change detection from level 11 to level 12 using the proposed and traditional map overlay methods. (**a**) The proposed method, (**b**) Traditional overlay method.

**Figure 11:**
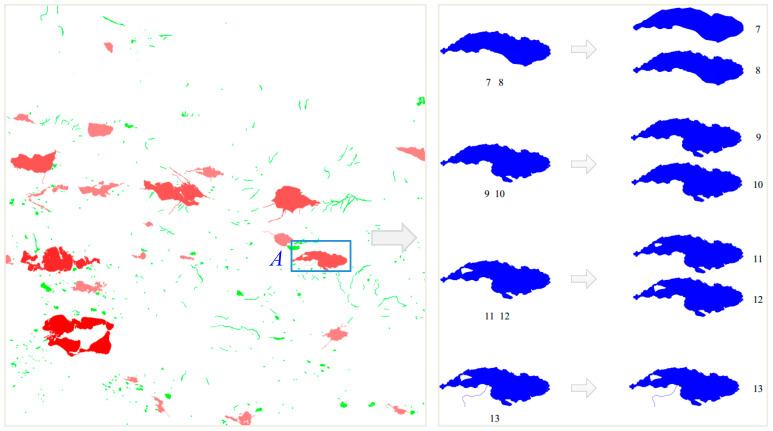
Hierarchical expression of change information. Lake *A* is a typical sample.

Figure 10a shows the results of change detection from level 11 to level 12 using the proposed method, where red represents the change type of appearance, black represents the change type of disappearance, green represents the type of morphology change 2, brown represents the type of derivative change 2, yellow represents the type of hole change 1, and orange red represents the type of hole change 3. The thresholds used for change type detection are as follows: $\Delta_{\mathrm{SI}}=\mathrm{threshold}\leq 20^{\circ }$, and $\Delta_{GC}=threshold\leq 100$. We also applied the map overlay [55] method for the change detection of this region. The corresponding results of change detection can be found in Figure 10b, where red represents the positive change of water areas, and black represents the negative change of water areas. Compared with the traditional map overlay method, two advantages of the proposed method should be addressed. (1) As shown in Figure 10, the proposed method can effectively detect many different types of changes, such as morphology change, hole change, and derivative change, while the traditional map overlay method can only divide the changes into two types, namely, positive and negative changes, as shown in the black box. (2) As shown in Figure 11 in the next subsection, the changes of water areas can be hierarchically expressed, which enables the reader to progressively acquire change information at different levels of detail, while the traditional map overlay method cannot achieve this.

(2) Hierarchical expression of change

In turn, a water area can be expressed in the body, piece, and slice layers by using the proposed hierarchical ideas. For example, consider the case of lake *A* marked in Figure 11.

In the body layer, the situation of global change is measured. As shown on the left side of Figure 11, the changes in the representative area value from level 7 to level 13 are displayed by gradient colors. The deeper the red color of the area is, the larger the fluctuation in the area change is. Green represents the lakes without increases and decreases in their area within a tolerance range. The figure shows that the larger the area of a lake is, the more powerful the fluctuation in the area change is. When the area of a lake is smaller, it is not easy to observe larger changes in the area. The areal variance in lake *A* in the box is larger. Therefore, the deeper the red color is, the larger the area fluctuation in lake *A* in the body layer is.

In the piece layer, the mutation information is expressed. As shown in Table 4, according to the calculation method proposed in this paper, the mutations in lake *A* are found at levels 8–9, 10–11, and 12–13, where the mutation types are morphology change 1, hole change 1, and morphology change 2, respectively. Therefore, the lakes at seven levels are divided into four groups. The change between groups is larger, but the change within one group is smaller.

In the slice layer, after the expression of lake *A* in the piece layer, the detailed changes in the objects in one group are expressed. As shown in the image on the right side of Figure 11, boundary generalization occurred in levels 7–8. There is no change in levels 9–10. Hole change 3 occurs in levels 11–12. The difference in each kind of change before and after the overall change is shown in Table 5.

## 5. Conclusions

During mental processes, humans usually solve problems by starting with the general scope and then focusing on the details hierarchically [34]. In this paper, we proposed the hierarchical change detection principle at multiple scales from the perspective of spatial cognition. We also classified water area changes, established a set of indicators and rules for the change detection of water areas in multi-scale raster maps, and determined the key technology for water extraction from tile maps. Compared with the traditional water change detection methods at the same or adjacent scales, the following conclusions can be drawn:

(1) The proposed change detection method of water areas contains more detailed change types and can even examine the changes generated from map generalization, while the traditional method cannot.

(2) The representation of water area changes is hierarchical, which enables readers to progressively acquire change information at different levels of detail. Thus, the cognitive burden caused by displaying too much information can be well avoided.

As the proposed method for the change detection of water areas is suitable for any binary image, it can be applied for other map sources or other locations when the corresponding map elements are extracted. For some open source tile maps that have data quality problems, such as OpenStreetMap, the proposed change detection method is very helpful for data quality improvement. For example, when volunteers upload map data to the OpenStreetMap database, the proposed method can be used to filter out inconsistent data with great changes and ensure the correctness of input data. However, indexes that are used to describe the change patterns in the body layer, such as the standard deviation in area, are relatively simple, and finding more effective and reasonable measure indexes is worthy of further consideration. Furthermore, because the collapse operation is usually applied to rivers with long and narrow shapes, the detection indicators and methods are not designed for the collapse of areal lakes. In addition, we only applied the tile map with regular color values as the experimental data, and complex data sources such as remote sensing images should be explored in the future. As time series change is also an important research topic, future research should focus on the seasonal changes of water areas using tile map data.

## Figures and Tables

**Table 1:** Change types.

Change Type	Before Change	After Change	Change Type	Before Change	After Change
no change	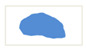	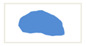	appearance	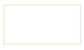	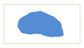
disappearance	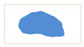	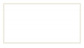	movement	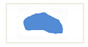	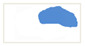
rotation	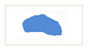	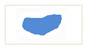	scaling	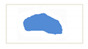	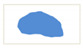
morphology change	1 append		hole change	1 appear	
	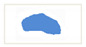	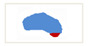		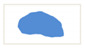	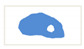
	2 distributary			2 disappear	
	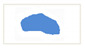	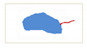		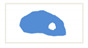	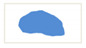
	3 shape change			3 structure change	
	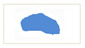	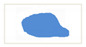		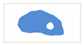	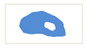
derivative change	1 merge		boundary generalization	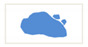	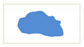
	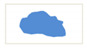	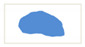			
	2 split				
	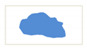	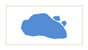			

**Table 2:** Detection rules of the change types.

Types	Operations	Relevant and Fixed	Relevant and Threshold	Rules
appearance and disappearance	null	*MT*	null	MT=1:0 or MT=0:1.
movement	null	$MT,\Delta_{L},\Delta_{A},$ $\Delta_{SI},HS$	$\Delta_{GC}$	MT=1:1; $\Delta_{GC}=threshold>0$; $\Delta_{L}=\Delta_{A}=\Delta_{SI}=HS=0$.
rotation	move until meeting centre and overlap	$MT,\Delta_{L},\Delta_{A},$ $\Delta_{SI},HS$	OD	MT=1:1; $\Delta_{L}=\Delta_{A}=\Delta_{SI}=HS=0$; $OD_{x}=threshold<100\%$; $OD_{y}=threshold<100\%$.
scaling	scaling to the same size and overlap	$MT,\Delta_{SI},\Delta_{GC},$ HS,OD,BOD	null	MT=1:1; $HS=\Delta_{SI}=\Delta_{GC}=0$; $OD_{x}=OD_{y}=BOD=100\%$
boundary generalization	overlap	MT,HS,BOD	$\Delta_{SI},\Delta_{GC}$	MT=1:1; HS=0; BOD=100%; $\Delta_{SI}=threshold$; $\Delta_{GC}=threshold$.
morphology change: append	overlap	MT,GT,HS	OD	MT=1:1; HS=0; $OD_{x}=100\%$; $OD_{y}\neq 100\%$; $GT_{y}=1$.
morphology change: distributary	overlap	MT,GT,HS	OD	MT=1:1; $OD_{x}=100\%$; $OD_{y}\neq 100\%$; $HS=GT_{y}=0$.
morphology change: shape change	overlap	MT ,HS	OD,BOD	MT=1:1; HS=0; $OD_{x}\neq 100\%$; $OD_{y}\neq 100\%$; BOD=threshold<100%.
derivative change: merge	null	*MT*	null	MT=m:1
derivative change: split	null	*MT*	null	MT=1:n
hole change: appear and disappear	null	MT,HS	null	MT=1:n; HS=1.
hole change: structure change	null	MT,HS	$\Delta_{L},\Delta_{A}$	MT=1:n; HS=1; $\Delta_{L}\neq 0$; $\Delta_{A}\neq 0$.

**Table 3:** Change type statistics.

	7–8	8–9	9–10	10–11	11–12	12–13	Total
appearance	0	0	0	130	357	28	515
disappearance	0	0	0	0	5	1	6
movement	0	0	0	0	0	0	0
rotation	0	0	0	0	0	0	0
scaling	0	0	0	0	0	0	0
boundary generalization	2	0	0	0	0	0	2
morphology change 1	0	2	3	0	0	5	10
morphology change 2	0	0	0	0	9	4	13
morphology change 3	0	2	1	0	0	0	3
derivative change 1	0	0	0	0	0	0	0
derivative change 2	0	0	0	7	2	0	9
hole change 1	2	0	0	6	18	0	26
hole change 2	0	0	0	0	0	0	0
hole change 3	0	1	0	0	6	16	23
Total	4	5	4	143	397	54	607

**Table 4:** Changes in the piece layer.

Level	8–9	10–11	12–13
Mutation type	morphology change 1	hole change 1	morphology change 2

**Table 5:** Changes in the slice layer.

Level	7–8	9–10	11–12	13
Change type	boundary generalization	no change	hole change 3	no change
difference	$\Delta_{SI}=9^{\circ }$	0	$\Delta_{A}=230pel$	0
